# 

**DOI:** 10.1192/bjb.2025.10165

**Published:** 2026-06

**Authors:** Dennis Ougrin

**Affiliations:** Professor of Child and Adolescent Psychiatry, Youth Resilience Unit, Wolfson Institute of Population Health, Queen Mary University of London, London, UK

**Figure:**
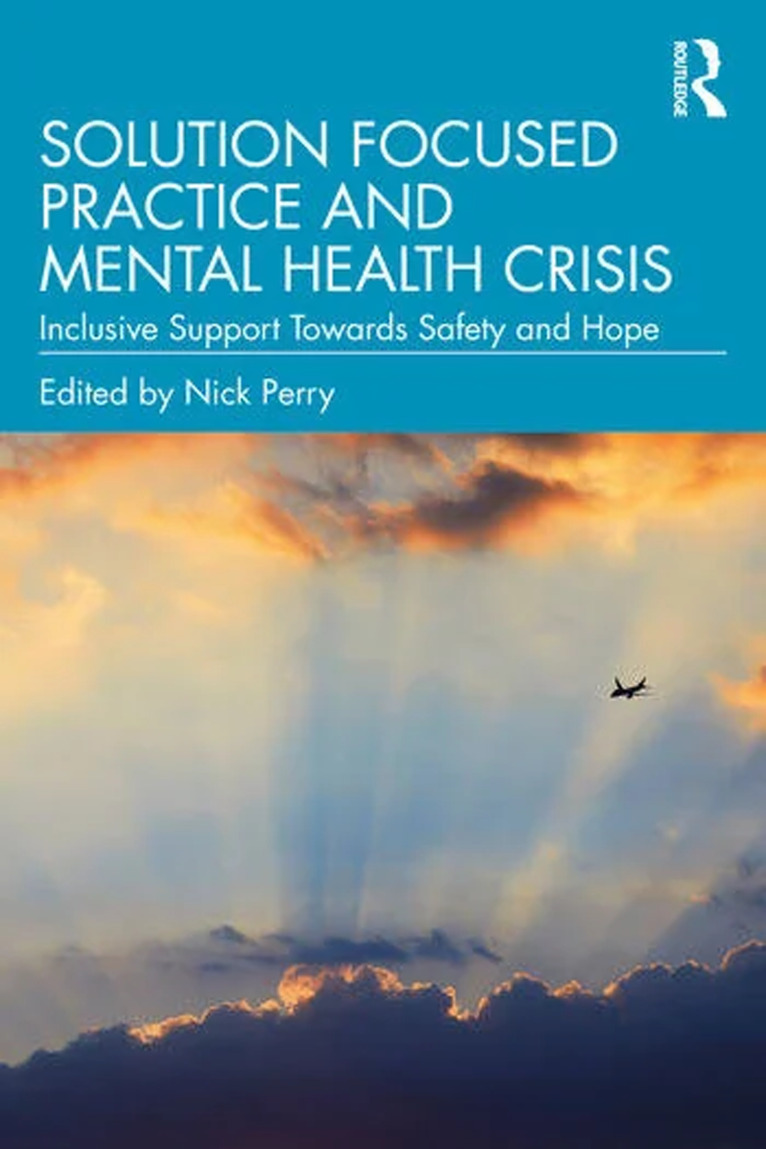

This highly accessible volume, edited by Nick Perry, makes a case for the use of solution focused practice (SFP) in mental health crisis care. Drawing on the expertise of psychiatrists, social workers, nurses, researchers and those with lived experience, the book offers a multidisciplinary perspective on how strengths-based, person-led and future-oriented conversations can be helpful in supporting people at times of acute distress.

Perry, an experienced Approved Mental Health Professional (AMHP), in his contribution argues that SFP can help mental health professionals uphold the guiding principles of the Mental Health Act – least restriction, empowerment and respect. He further demonstrates how SFP can support anti-racist practice in high-stakes statutory contexts.

The book’s breadth is impressive: contributors explore SFP’s use in emergency departments, police custody, statutory child and adolescent services and AMHP decision-making. Clinical insights are balanced with theory, including a neurobiological perspective from Adam Froerer and a comprehensive conceptual framework by Lauren Jerome.

Particularly powerful are the anonymised practice stories showing how SFP helps uncover patients’ resources and networks – essential when supporting people from minoritised communities who are disproportionately subjected to coercive interventions.

Despite these strengths, the book could do more to acknowledge the limited high-quality empirical evidence for SFP in general and for its use in crisis settings in particular. While large-scale trials such as those described in the chapter by Rose McCabe and colleagues are in progress, the current evidence base remains largely qualitative or anecdotal. Readers looking for robust comparative data – especially in relation to more established interventions – may find this a limitation.

Nonetheless, this volume succeeds as both an inspiring introduction and a practical guide. It challenges the reader to imagine a more collaborative, hopeful and culturally responsive approach to crisis care. For mental health professionals and policy leaders alike, it offers a clear and humane alternative to risk-driven, deficit-based models.

